# Sublethal treatment with plasma-activated medium induces senescence-like growth arrest of A549 cells: involvement of intracellular mobile zinc

**DOI:** 10.3164/jcbn.19-17

**Published:** 2019-06-11

**Authors:** Hirokazu Hara, Mari Kobayashi, Moe Shiiba, Tetsuro Kamiya, Tetsuo Adachi

**Affiliations:** 1 Laboratory of Clinical Pharmaceutics, Gifu Pharmaceutical University, 1-25-4 Daigaku-nishi, Gifu 501-1196, Japan

**Keywords:** plasma-activated medium, reactive oxygen species, zinc, growth arrest, p53

## Abstract

Plasma-activated medium (PAM) is a solution produced by exposing a liquid medium to non-thermal atmospheric pressure plasma (NTAPP). A number of reactive molecules, such as reactive oxygen species and reactive nitrogen species, are contained in PAM. Therefore, exposure to high doses of PAM results in cell death. We previously demonstrated that intracellular zinc (Zn^2+^) serves as an important mediator in PAM-induced cell death; however, the effects of sublethal treatment with PAM on cell functions are not fully understood. In the present study, we found that sublethal PAM treatment suppressed cell proliferation and induced senescence-like changes in lung adenocarcinoma A549 cells. Cell cycle analysis revealed that PAM induced cell cycle arrest at the G2/M phase. PAM increased the level of intracellular free Zn^2+^ and the Zn^2+^ chelator TPEN counteracted PAM-induced growth suppression, suggesting that Zn^2+^ functions in PAM-induced growth suppression. In addition, sublethal treatment with PAM induced phosphorylation of ATM kinase, accumulation of p53 protein, and expression of p21 and GADD45A, which are known p53 target genes, in a Zn^2+^-dependent manner. These results suggest that the induction of growth arrest and cellular senescence by sublethal PAM treatment is mediated by Zn^2+^-dependent activation of the ATM/p53 pathway.

## Introduction

Non-thermal atmospheric pressure plasma (NTAPP) is an ionized gas. NTAPP has been reported to be promising for use in the medical field, including in cancer therapy,^(1–3)^ blood coagulation,^(4)^ and wound healing.^(5,6)^ In particular, as NTAPP selectively kills cancer cells, its applicability to cancer therapy has been highly investigated. NTAPP interacts with liquids, such as culture medium surrounding cells, resulting in the generation of reactive oxygen species (ROS) and reactive nitrogen species (RNS) in the liquids.^(7)^ These reactive species have been reported to participate in several biological effects of NTAPP. Plasma-activated medium (PAM) is a solution produced by exposing a liquid medium to NTAPP. Indirect irradiation using PAM exerts cytotoxic effects against tumor cells as well as direct NTAPP irradiation does. As such, PAM is thought to be a useful anticancer therapeutic tool. We also previously reported that PAM induces cancer cell death associated with energy failure.^(8,9)^

Zinc (Zn^2+^), an essential trace element, is required for the structural stability and function of a large number of proteins, including transcription factors and metalloenzymes. On the other hand, Zn^2+^ is a very toxic metal and almost all intracellular Zn^2+^ is bound to proteins through coordination of cysteine residues. However, as Zn^2+^/cysteine clusters are redox-sensitive, ROS/RNS react with the clusters to liberate Zn^2+^ and the liberated Zn^2+^ then induces cell injury.^(10–12)^ Oxidative stress-induced cell death was reported to be suppressed in the presence of the Zn^2+^ chelator *N*, *N*, *N*', *N*'-tetrakis (2-pyridylmethyl) ethylenediamine (TPEN).^(13)^ The mechanism by which the liberated Zn^2+^ induces cell death is not fully understood; however, Zn^2+^ was found to inhibit glyceraldehyde-3-phosphate dehydrogenase (GAPDH), a rate-limiting enzyme in glycolysis, and the mitochondrial electron transport chain, resulting in energy failure and generation of ROS.^(14)^ We previously demonstrated that PAM exposure raises the level of free Zn^2+^ in the cytosol and nucleus, and causes mitochondrial dysfunction in neuroblastoma SH-SY5Y cells.^(8,9)^

Several reports demonstrated that the exposure of cells to PAM induces severe cell injury, whereas sublethal NTAPP irradiation (direct method) and PAM treatment (indirect method) protects cells from oxidative stress. Indeed, we and others reported that sublethal treatments activate the Keap1/Nrf2 system, which plays an important role in adaptive responses to oxidative stress, and induce antioxidative genes.^(15,16)^ However, which biological function sublethal treatment with PAM causes is unclear. A sublethal amount of hydrogen peroxide is known to induce growth arrest and cellular senescence. As mentioned above, PAM contains various reactive molecules such as hydrogen peroxide and nitrite. Thus, the purpose of this study was to examine the effects of sublethal PAM treatment on cell proliferation of human lung adenocarcinoma epithelial A549 cells and to elucidate the involvement of intracellular Zn^2+^ in PAM-induced growth suppression.

## Materials and Methods

### Materials

The ATM inhibitor KU-55933 was purchased from Wako Pure Chemicals (Osaka, Japan). TPEN was purchased from Dojindo (Kumamoto, Japan). Anti-p53 antibody was purchased from Merck (Darmstadt, Germany). Anti-ATM, anti-phospho ATM, anti-phospho p53 (Ser 15), and γH2AX antibodies were purchased from Cell Signaling Technology (Danvers, MA). 3-(4,5-Dimethylthiazol-2-yl)-2,5-diphenyltetrazolium bromide (MTT) was purchased from Sigma Aldrich (St. Louis, MO). FluoZin-3 AM was purchased from Thermo Fischer Scientific (Waltham, MA).

### Preparation of plasma-activated medium (PAM)

 PAM was prepared as described in our previous report.^(9)^ Briefly, we used an irradiation system that consists of a power controller/gas flow regulator, an argon (Ar) gas cylinder, and a plasma source head (PN-120 TPG; NU Global, Nagoya, Japan) in this study. DMEM without pyruvate (D5796; Sigma Aldrich) in a 3.5-cm-diameter dish was irradiated with NTAPP for 3 min at a flow rate of Ar gas of 2 L/min. The distance between the plasma source and the surface of the medium was fixed at 3 mm.

### Cell culture

Human lung adenocarcinoma epithelial A549 cells were cultured in growth medium (DMEM supplemented with 10% fetal calf serum (FCS), 100 units/ml of penicillin G, and 0.1 mg/ml of streptomycin) in a humidified 5% CO_2_/95% air incubator at 37°C.

### Cell count

A549 cells were seeded in a 24-well plate (1.0 × 10^5^ cells/well). The next day, cells were treated with serum-free DMEM (500 µl) containing of PAM (75 µl) for 1 h. The treatment with PAM was terminated by replacing PAM-containing DMEM with the growth medium, and then cells were further cultured for 12, 24, 48, or 72 h. After trypsinization, the number of cells was counted.

### Senescence-associated β-galactosidase assay

A549 cells were seeded in a 3.5-cm-diameter dish (1.0 × 10^5^ cells/dish). The next day, cells were treated with serum-free DMEM (1.5 ml) containing PAM (250 µl) for 1 h, followed by culture in the growth medium for another 72 h. β-Galactosidase activity was detected using the Senescence β-Galactosidase Staining Kit (Cell Signaling Technology).

### Cell growth assay

A549 cells were seeded in a 96-well plate (1.2 × 10^4^ cells/well). The next day, cells were treated with serum-free DMEM (100 µl) containing varying volumes of PAM for 1 h, followed by culture in the growth medium for another 20 h. Cell growth was measured using the MTT assay. The experiments for cell growth were carried out in quadruplicate. The results are expressed as percentages relative to untreated cells.

### Lactate dehydrogenase (LDH) cytotoxicity assay

 A549 cells were seeded in a 96-well plate (1.2 × 10^4^ cells/well). The next day, cells were treated with serum-free DMEM (100 µl) containing varying concentrations of PAM for 1 h, followed by culture in the growth medium for another 20 h. LDH activity in the conditioned medium was measured using the LDH-Cytotoxic Test (Wako Pure Chemical). The experiments were carried out in quadruplicate. The results are expressed as the fold change relative to untreated cells.

### Cell cycle analysis

A549 cells were seeded in a 6-cm-diameter dish (1.0 × 10^6^ cells/dish). The next day, cells were treated with serum-free DMEM (3 ml) containing PAM (500 µl) for 1 h in the presence or absence of TPEN (10 µM), followed by culture in the growth medium for another 24 h. Cells were trypsinized, and collected by centrifuging (1,500 rpm, 5 min). The cells were fixed with 70% ethanol for 2 h, treated with RNase (50 µg/ml) for 60 min, and stained with propidium iodide (PI, 5 µg/ml). The stained cells were analyzed using BD FACSVerse (BD Bioscience, San Jose, CA).

### Zn^2+^ imaging

Zn^2+^ imaging was performed as described in our previous report.^(9)^ Briefly, A549 cells were seeded in 3.5-cm-diameter dish (1 × 10^5^ cells/dish). The cells loaded with FluoZin-3 were treated with PAM. Time-lapse fluorescence imaging of live cells was performed in 30-s intervals for 20 min using a confocal laser fluorescence microscope (LSM700, Carl Zeiss, Germany). The images were quantified using ZEN software (Carl Zeiss).

### Western blotting

Western blotting was performed as described in our previous report.^(9)^ A549 cells were seeded in a 6-cm-diameter dish (1.0 × 10^6^ cells/dish). After the treatment, cells were washed twice with ice-cold PBS and lysed using 150 µl of the lysis buffer. Aliquots of the prepared samples were separated by SDS polyacrylamide gel electrophoresis (SDS-PAGE) on a 12% polyacrylamide gel and transferred onto a polyvinylidene difluoride (PVDF) membrane. The membrane was sequentially incubated with each primary antibody (1:3,000), biotin-conjugated secondary antibody (1:3,000), and ABC reagents (Vector Laboratories, Burlingame, CA) (1:5,000). Finally, proteins were detected using Super-Signal West Pico Chemiluminescent Substrate (Thermo Fisher Scientific) or ImmunoStar LD (Wako Pure Chemical), and imaged using ChemDoc Touch (Bio-Rad, Hercules, CA).

### Reverse transcription-PCR (RT-PCR)

A549 cells were seeded in a 6-cm-diameter dish (1.0 × 10^6^ cells/dish). The next day, cells were treated with PAM (0.5 ml) for 1 h in the presence or absence of TPEN (10 µM), followed by culture in the growth medium for another 7 h. Total RNA was extracted from the treated cells with TRIzol reagent (Invitrogen, Carlsbad, CA). First strand cDNA was synthesized from 1 µg of total RNA. Aliquots of the cDNA solution (1 µl) were amplified using the following specific primers: human p21 (forward primer, 5'-CTGGGGATGTCCGT CAGAAC-3'; reverse primer, 5'-TGAGAGTCTCCAGGTCC ACC-3'), human GADD45A (forward primer, 5'-CGAGAACGA CATCAACATCCTG-3'; reverse primer, 5'-TTGATCCATGTA GCGACTTTCC-3'), human ZnT1 (forward primer, 5'-GCCAAT ACCAGCAACTCCAAC-3'; reverse primer, 5'-TCCAGCCCT ATCTTCTTCCAG-3'), human ZIP1 (forward primer, 5'-GTT CCCACTGCAAGAGTTCATC-3'; reverse primer, 5'-GAGAAC ACCAGTACACAGGCAC-3'), and human GAPDH (forward primer, 5'-GAAGGTGAAGGTCGGAGTC-3'; reverse primer, 5'-CAAAGTTGTCATGGATGACC-3'). PCR was carried out as follows: initially 2 min at 94°C, followed by 28 (p21), 30 (GADD45A, ZnT1, and ZIP1), or 18 (GAPDH) cycles of 40 s at 94°C, 40 s at 60°C, and 1 min at 72°C. Aliquots of the PCR mixtures were separated on 2% agarose gel and stained with ethidium bromide.

### Statistics

Data was analyzed using ANOVA followed by the *post hoc* Bonferroni or Holm method. A *p* value less than 0.05 was considered significant.

## Results

### Effects of sublethal treatment with PAM on cell proliferation

**Fig. 1:**
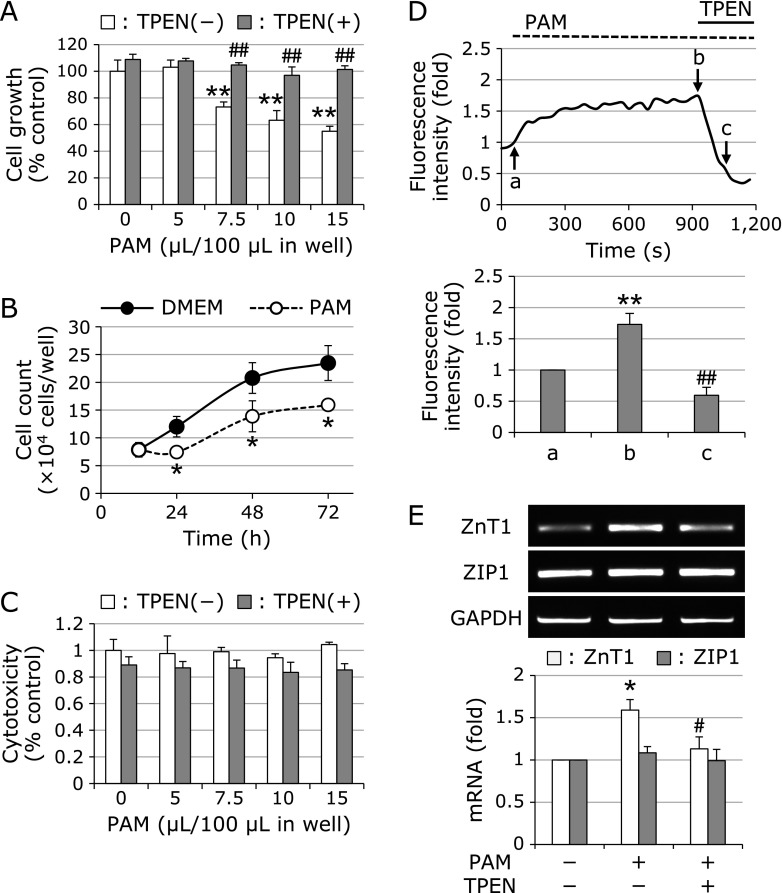
Zn^2+^-dependent growth suppression of A549 cells sublethally treated with PAM. (A) MTT assay. A549 cells were treated with varying doses of PAM for 1 h in the presence or absence of TPEN (10 µM), and then cultured in the growth medium for another 20 h. Values are means ± SD from four separate cultures. **p<0.01 (vs untreated cells); ^##^*p*<0.01 (vs without TPEN). (B) Cell count. A549 cells were exposed to PAM (75 µl/500 µl) for 1 h, and then cultured in the growth medium for another 12, 24, 48, or 72 h. Values are means ± SD from four separate cultures. *p<0.05 (vs control). (C) LDH assay. A549 cells were exposed to varying doses of PAM for 1 h in the presence or absence of TPEN (10 µM), followed by culture in the growth medium for another 20 h. Values are means ± SD from four separate cultures. (D) PAM-induced Zn^2+^ liberation. A549 cells loaded with FluoZin-3 AM were treated with PAM (500 µl/1.5 ml). Fifteen minutes after the addition of PAM, TPEN was added to the culture at a final concentration of 10 µM. Fluorescence was detected using a confocal microscope. Statistical analysis of the fluorescence intensity is shown below. a, PAM addition; b, before TPEN addition; c, after TPEN addition. **p<0.01 (vs a); ^##^*p*<0.01 (vs b). (E) Effects of PAM on expression of Zn^2+^ transporters. A549 cells were treated with PAM (500 µl) for 1 h in the presence or absence of TPEN (10 µM), and then cultured in the growth medium for another 7 h. After treatment, RT-PCR was performed. Values are the means ± SEM from three separate cultures. *p<0.05 (vs untreated cells); ^#^*p*<0.05 (vs PAM-treated cells).

PAM-triggered cellular responses vary with differences in the intensity of PAM treatment (e.g., exposure time and dosage).^(15,17,18)^ We previously reported that long-term exposure (6 h) of A549 cells to PAM induces marked cell injury.^(1)^ On the other hand, cellular responses induced by sublethal treatment with PAM are unclear. First, to examine the effects of sublethal PAM treatment on cell proliferation, A549 cells were treated with low doses of PAM for 1 h, followed by culture in growth medium for 20 h. The dosage of PAM (15 µl/100 µl DMEM) was equal to approximately 100 µM H_2_O_2_. After treatment, we evaluated cell growth using the MTT assay. As shown in Fig. 1A, PAM dose-dependently inhibited cell proliferation. Consistent with this proliferation assay, sublethal treatment with PAM reduced the number of cells (Fig. 1B). However, LDH release from cells exposed to PAM was not observed (Fig. 1C), suggesting that PAM did not cause cytotoxicity under these experimental conditions.

### Involvement of intracellular Zn^2+^ in sublethal PAM-induced growth suppression

In our previous reports, intracellular Zn^2+^ was found to play an important role in PAM-triggered cellular responses.^(8,9)^ To clarify the involvement of intracellular Zn^2+^ in sublethal PAM-induced growth suppression of A549 cells, we examined the effects of the Zn^2+^ chelator TPEN on this phenomenon. A549 cells were exposed to PAM for 1 h in the presence or absence of TPEN, followed by culture in the growth medium for another 20 h. The chelator prevented PAM-induced growth inhibition and did not induce cell injury (Fig. 1A and C). As such, we ascertained whether PAM increases the level of intracellular free Zn^2+^ in A549 cells. The intracellular free Zn^2+^ levels were measured by the fluorescence Zn^2+^ indicator, FluoZin-3. An increased level of intracellular Zn^2+^ caused by PAM exposure was observed (Fig. 1D). It has been reported that intracellular Zn^2+^ levels are regulated by several Zn^2+^ transporters.^(19)^ We investigated the effects of PAM on expression of ZnT1 (Zn^2+^ exporter) and ZIP1 (Zn^2+^ importer) in A549 cells. PAM induced *ZnT1* mRNA expression, whereas it did not affect *ZIP1* mRNA expression (Fig. 1E).

### Sublethal treatment with PAM induces G2/M growth arrest and senescence-like changes

**Fig. 2:**
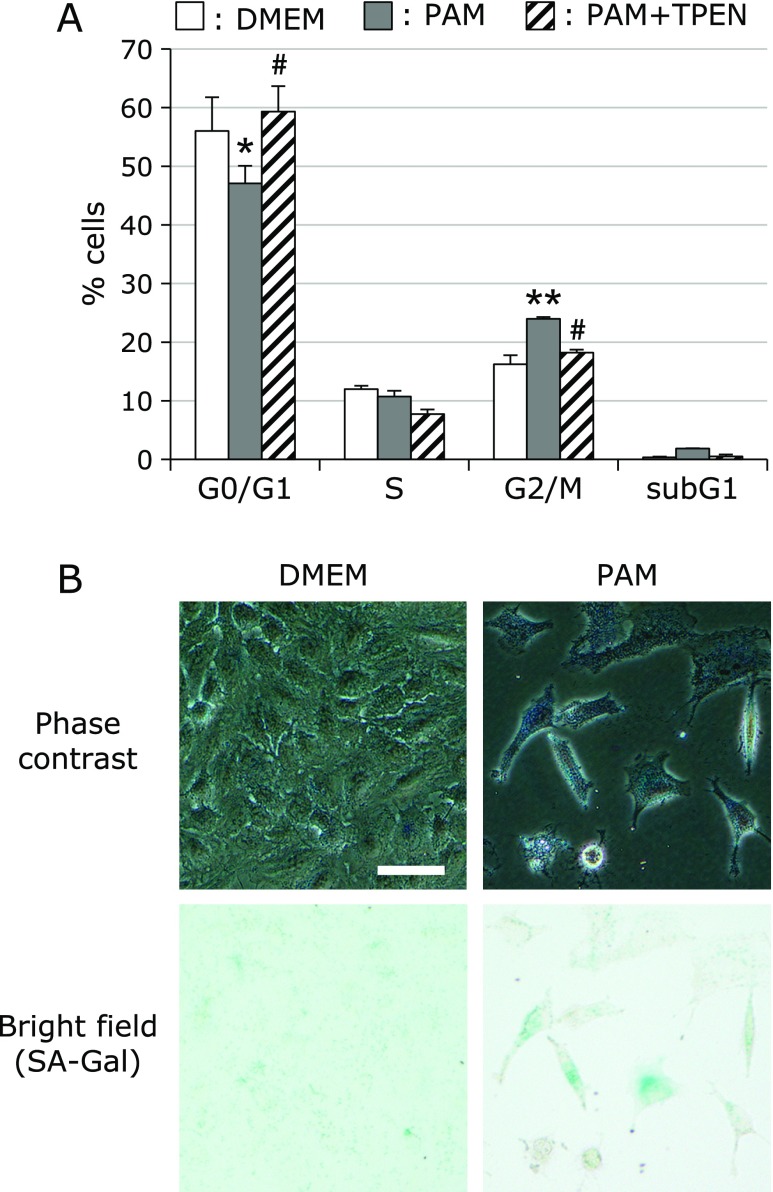
Sublethal treatment with PAM induces G2/M arrest and senescence-like changes. (A) Cell cycle analysis. A549 cells were treated with PAM (500 µl/3 ml) for 1 h, and then cultured in the growth medium for another 24 h. After treatment, cells were fixed and stained with PI, followed by flow cytometry analysis. Values are the means ± SEM from four separate cultures. *p<0.05, **p<0.01 (vs DMEM); ^#^*p*<0.01 (vs PAM alone). (B) β-Galactosidase activity. A549 cells were treated with PAM (250 µl/1.5 ml) for 1 h, and then cultured in the growth medium for another 72 h. After treatment, cells were fixed and stained with X-gal. Bar = 50 µm.

To investigate the effects of sublethal PAM on cell cycle progression, we analyzed the cell cycle using flow cytometry. As shown in Fig. 2A, PAM reduced the percentage of cells in the G0/G1 phase, but increased that of cells in the G2/M phase. These changes were counteracted by TPEN. PAM also slightly increased the percentage of cells in subG1.

Low levels of oxidative stress are known to prevent cell proliferation and induce cellular senescence. To examine whether sublethal treatment with PAM promotes cellular senescence, we measured the activity of senescence-associated β-galactosidase (SA β-Gal), which is widely used as a marker cellular senescence. After treatment of A549 cells with sublethal PAM for 1 h, cells were further cultured in the growth medium for 72 h, followed by X-gal staining. SA β-Gal-positive cells were observed in PAM-treated cultures (Fig. 2B).

### PAM-induced accumulation of p53 protein

**Fig. 3:**
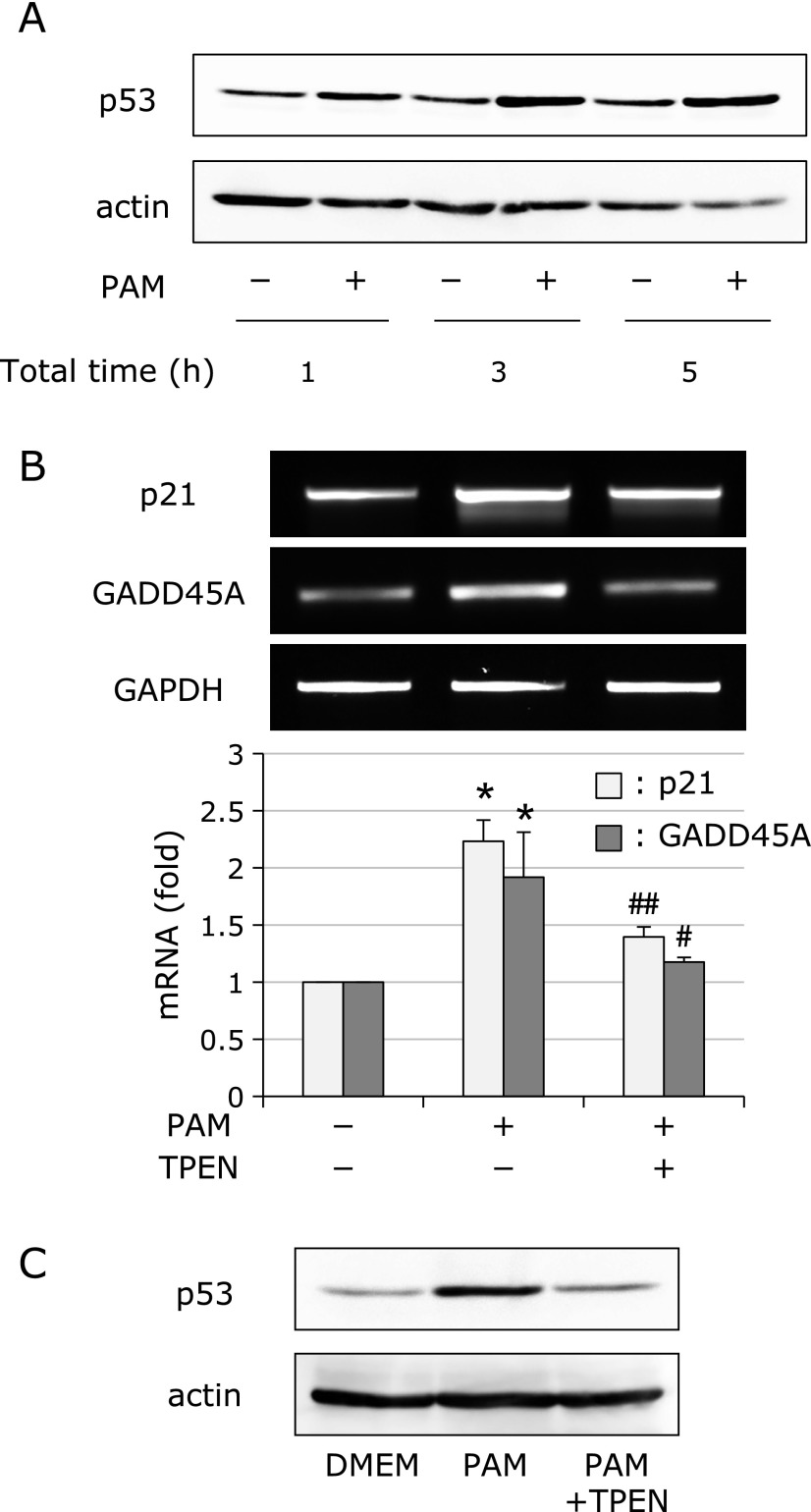
Effects of PAM on activation of the p53 signaling pathway. (A) PAM-induced accumulation of p53 protein. A549 cells were treated with PAM (500 µl) for 1 h, and then cultured in the growth medium for another 2 or 4 h. After treatment, Western blotting analysis was performed. (B) Effects of PAM on expression of p53 target genes. A549 cells were treated with PAM (500 µl) for 1 h in the presence or absence of TPEN (10 µM), and then cultured in the growth medium for another 7 h. After treatment, RT-PCR was performed. Values are the means ± SEM from four separate cultures. *p<0.01 (vs untreated cells); ^#^*p*<0.05, ^##^*p*<0.01 (vs PAM-treated cells). (C) Effects of TPEN on p53 protein accumulation. A549 cells were treated with PAM (500 µl) for 1 h in the presence or absence of TPEN (10 µM), and then cultured in the growth medium for another 4 h. After treatment, Western blotting analysis was performed.

The tumor suppresser protein p53 is well known to play an important role in cell cycle arrest.^(20)^ In general, p53 is rapidly degraded under normal conditions; however, stress inhibits its degradation and promotes the accumulation of p53 protein. We therefore examined the effects of sublethal PAM treatment on its expression. A549 cells were treated with PAM for 1 h, followed by culture in growth medium for another 2 and 4 h. As shown in Fig. 3A, PAM time-dependently raised the levels of p53 protein. In addition, we examined the effects of PAM on the expression of p21 and GADD45A, which are known downstream targets of p53. As expected, PAM induced expression of both genes (Fig. 3B). Moreover, PAM-induced accumulation of p53 and expression of *p21* and *GADD45A* mRNA were suppressed in the presence of TPEN (Fig. 3B and C).

### PAM-induced activation of ATM kinase

**Fig. 4:**
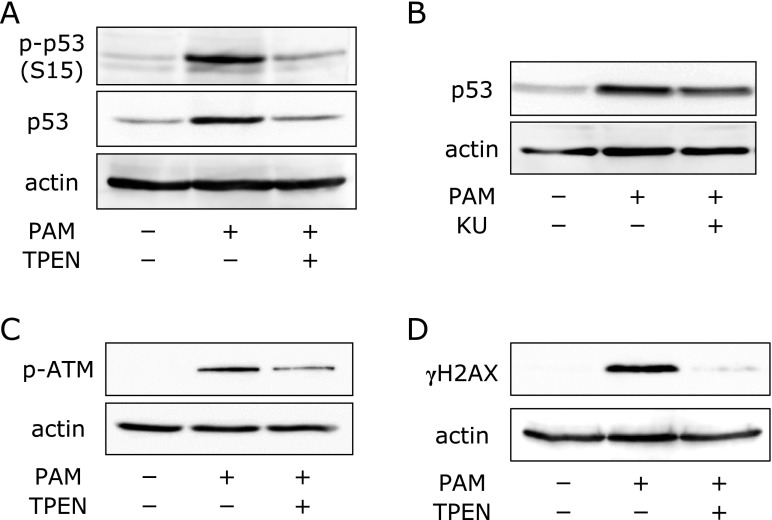
Involvement of Zn^2+^ in PAM-induced activation of the ATM/p53 pathway. (A) PAM-induced p53 phosphorylation. A549 cells were treated with PAM (500 µl/3 ml) for 1 h in the presence or absence of TPEN (10 µM), followed by culture in the growth medium for another 4 h. After treatment, Western blotting analysis was performed. (B) Effects of KU-55933 on PAM-induced p53 accumulation. A549 cells were treated with PAM (500 µl/3 ml) for 1 h in the presence or absence of KU-55933 (KU, 50 µM), followed by culture in the growth medium for another 4 h. After treatment, Western blotting analysis was performed. (C) PAM-induced ATM phosphorylation. A549 cells were treated with PAM (500 µl/3 ml) for 30 min in the presence or absence of TPEN (10 µM). After treatment, Western blotting analysis was performed. (D) PAM-induced γH2AX accumulation. A549 cells were treated with PAM (500 µl/3 ml) for 1 h in the presence or absence of TPEN (10 µM). After treatment, Western blotting analysis was performed.

ATM kinase, a key kinase responsive for the DNA repair, has been reported to be activated by several stimuli such as oxidative stress and genotoxic drugs.^(21)^ Activated ATM elicits p53 phosphorylation, promotes its stabilization, and initiates stress responses, including growth arrest and apoptosis. We thus investigated whether sublethal treatment with PAM activates the ATM/p53 signaling pathway. A549 cells were treated with PAM for 1 h, followed by culture in growth medium for another 4 h. Phosphorylation of p53 at Ser15 was observed (Fig. 4A). The ATM inhibitor KU-55933 suppressed PAM-induced accumulation of p53 (Fig. 4B). In addition, we investigated the alteration in phosphorylation status of ATM by Western blotting. PAM rapidly induced ATP phosphorylation (Fig. 4C). DNA damage induced by stress is known to induce ATM activation. Lastly, to examine whether sublethal treatment with PAM causes DNA damage, we analyzed γH2AX, a DNA damage marker. Increased levels of γH2AX (phosphorylated H2AX) were observed in PAM-treated cells (Fig. 4D). TPEN suppressed the PAM-induced γH2AX increase and activation of the ATM/p53 pathway (Fig. 4A, C and D).

## Discussion

In this study, we found that sublethal treatment with PAM caused G2/M growth arrest and senescence-like changes in A549 cells. PAM-induced growth arrest was regulated through activation of the ATM-p53 pathway. The addition of PAM to cells rapidly increased the level of intracellular free Zn^2+^, whereas TPEN counteracted the PAM-induced activation of the ATM-p53 pathway and subsequent growth arrest. These results suggest that Zn^2+^ signals initiated by PAM function in PAM-induced growth inhibition.

The susceptibility to PAM varies among different cell types. We previously demonstrated that SH-SY5Y cells are more susceptible to PAM toxicity than skin fibroblasts, and that the levels of Zn^2+^ liberated by PAM exposure are lower in less PAM-susceptible fibroblasts than in SH-SY5Y cells.^(8,9)^ As such, intracellular free Zn^2+^ levels are likely to affect PAM susceptibility. In the present study, we found that sublethal treatment with PAM suppressed proliferation of A549 cells in a Zn^2+^-dependent manner. This suggests that intracellular Zn^2+^ also serves as an important mediator in the PAM-induced growth arrest of A549 cells. As PAM did not cause cytotoxicity in A549 cells under these experimental conditions, the liberated Zn^2+^ levels may not be too high. Moreover, we demonstrated here that PAM induced *ZnT1* mRNA expression. Zn^2+^ is reported to promote *ZnT1* gene expression.^(22)^ Thus, these results strongly support the view that PAM treatment increased intracellular free Zn^2+^.

The majority of intracellular Zn^2+^ is bound to proteins through Zn^2+^/cysteine coordination. Therefore, intracellular free Zn^2+^ levels are very low in general. Although Zn^2+^ is a redox-inert metal, Zn^2+^/cysteine clusters are redox-sensitive. Therefore, ROS/RNS react with the clusters to promote the liberation of Zn^2+^ from different proteins such as metallothionein and zinc-finger transcription factors.^(11,23,24)^ As PAM contains many reactive species, including hydrogen peroxide and nitrite, these reactive molecules likely play a role in the PAM-induced increase of the intracellular free Zn^2+^ level. Indeed, we previously demonstrated that the antioxidant *N*-acetylcysteine counteracts the increase in intracellular free Zn^2+^ by PAM in SH-SY5Y cells. However, it is currently unclear which reactive molecules contained in PAM provoke Zn^2+^ liberation. Hydrogen peroxide and nitric oxide have been reported to promote Zn^2+^ release from intracellular Zn^2+^ stores in several cell types.^(12,25–27)^ Knoch *et al.*^(28)^ demonstrated that peroxynitrite triggers Zn^2+^ liberation, leading to Zn^2+^-dependent neuronal cell death. NTAPP irradiation generates ROS/RNS to produce peroxynitrite, which reacts with protein and causes protein nitration. Although it is unclear whether the treatment of cells with PAM generates peroxynitrite, we recently reported that PAM exposure causes the production of nitrotyrosine.^(29)^ Therefore, PAM-induced cysteine *S*-nitrosylation may play a role in Zn^2+^ liberation in A549 cells.

NTAPP irradiation was demonstrated to induce growth arrest at the G2/M phase.^(30)^ We also found that sublethal treatment with PAM resulted in G2/M arrest in A549 cells. A sublethal dose of hydrogen peroxide was found to induce cell cycle arrest at G1 and G2/M phases, and senescence-like changes in several cell types.^(31–34)^ As the concentration of hydrogen peroxide in PAM used in this study was approximately 100 µM (data not shown), ROS contained in PAM likely function in the induction of G2/M phase arrest. Furthermore, the p53 signaling pathway plays a central role in hydrogen peroxide-induced cell cycle arrest. In the present study, PAM induced the accumulation of p53 protein, and the expression of *p21* and *GADD45A* mRNA. p21, which is a cyclin-dependent kinase inhibitor, is widely known to regulate the cell cycle at the G1 checkpoint, whereas some reports have demonstrated that this molecule is involved in regulation of G2/M arrest.^(35,36)^ GADD45A has also been reported to mediate G2/M arrest and cellular senescence in a p53-dependent manner.^(37)^ Therefore, we consider that PAM-induced growth arrest is regulated by p53-dependent activation of p21 and GADD45A. In addition, TPEN prevented PAM-induced p53 activation, and subsequent *p21* and *GADD45A* mRNA induction. These results suggest that the liberated Zn^2+^ plays a role in the PAM-induced activation of p53. Indeed, several reports demonstrated that Zn^2+^ is closely related to the p53 signaling pathway. For example, Lin *et al.*^(38)^ found that ROS/RNS trigger intracellular Zn^2+^ release and activates ERK/GSK-3β/p53 signaling in a Zn^2+^-dependent manner in ischemic cardiomyocyte injury. In addition, we and others reported that Zn^2+^ itself stimulates p53 protein accumulation.^(39,40)^ Supplementation of Zn^2+^ increases the level of GADD45 protein and decreases that of Cdk1-Cyclin B1 complexes, leading to G2/M arrest.^(37)^

Ionizing radiation-induced double-strand DNA breaks (DSB) were found to provoke activation of ATM, a DNA damage response kinase.^(21)^ Activated ATM phosphorylates Ser15 of p53 and inhibits its degradation, leading to the activation of p53 signaling. In the present study, sublethal treatment with PAM increased the level of γH2AX and stimulated ATM activation in A549 cells. In addition, as TPEN suppressed this PAM-induced ATM activation, intracellular Zn^2+^ likely functions in this event. These results suggest that the ATM-p53 pathway is Zn^2+^-dependently activated by a sublethal dose of PAM. However, the mechanism by which liberated Zn^2+^ activates ATM remains unclear. NTAPP irradiation and PAM treatment were reported to cause DNA damage. Indeed, DNA damage occurred in A549 cells under our experimental conditions. DSB stimulate recruitment of the Mre11-Rad50-Nbs1 (MRN) complexes to the DSB site and the complexes in turn cause ATM activation.^(41)^ Of note, the formation of the complexes was demonstrated to be mediated by Zn^2+^.^(42)^ We previously revealed that PAM-induced Zn^2+^ liberation occurs in the nucleus. Therefore, the liberated Zn^2+^ may promote the formation of the complexes and lead to ATM activation. On the other hand, hydrogen peroxide directly activates ATM through oxidation of a specific cysteine residue in the absence of DNA damage.^(43)^ In addition, Zn^2+^ is known to inhibit complex I of the electron transport chain and activate NADPH oxidase, leading to ROS generation. Therefore, the possibility that Zn^2+^-induced ROS generation plays a role in ATM activation cannot be excluded. Further studies are needed to clarify the precise mechanism by which the liberated Zn^2+^ activates the ATM/p53 pathway.

In conclusion, we demonstrated that sublethal PAM treatment suppresses cell proliferation and causes senescence-like changes, and that intracellular Zn^2+^ acts as an important mediator in this phenomenon. Recently, increased cytoplasmic calcium caused by NTAPP-irradiation was found to be closely related to the induction of cellular senescence in melanoma cells.^(44)^ These findings suggest that the dynamics of intracellular metal ions in PAM-treated cells need to be elucidated in order to further understand the diverse PAM-induced cellular functions.
